# DYNAMICS OF HUMAN MUCOSAL-ASSOCIATED INVARIANT T CELL REPERTOIRES ACROSS THE HUMAN LIFE SPAN

**DOI:** 10.1093/geroni/igz038.2826

**Published:** 2019-11-08

**Authors:** Liyen Loh, Martha Lappas, Jane Crowe, Paul G Thomas, Sidonia Eckle, Dale I Godfrey, Katherine Kedzierska, Jeremy C Crawford

**Affiliations:** 1 Department of Microbiology and Immunology, The University of Melbourne at the Peter Doherty Institute or Infection and Immunity, Melbourne, Australia; 2 Department of Microbiology and Immunology, University of Melbourne, at the Peter Doherty Institute for Infection and Immunity, Melbourne, Victoria, Australia; 3 Deepdene Surgery, Deepdene, Victoria, Australia; 4 Department of Immunology, St Jude Children’s Research Hospital, Memphis, Tennessee, United States

## Abstract

Mucosal-associated invariant T (MAIT) cells are innate-like lymphocytes and are important for immune responses against bacterial and viral infections. While MAIT cells are known to undergo marked numerical changes with age in humans, our understanding of how these cells alter during these different phases across the human lifespan is largely unknown. Here we investigated MAIT cells from umbilical cord, children, young-adults and elderly. Functional analyses across 18-90 y/o adults showed that their MR1-dependent polyfunctionality was robust throughout old age. Strikingly, elderly MAIT cells displayed upregulated basal inflammatory cytokines, which were reduced to the level of young-adult MAIT cells in the absence of the aged environment. T cell receptor αβ analyses of MAIT cells across the human lifespan showed narrowing with age and large clonal TCRαβ expansions in elderly. These data suggest that MAIT cells in the elderly display remarkable plasticity, highlighting MAIT cells as key players in aged immune responses.

